# Correlation of Serum Cardiac Markers with Acute Decompensating Events in Liver Cirrhosis

**DOI:** 10.1155/2020/4019289

**Published:** 2020-09-24

**Authors:** Miaomiao Li, Zeqi Guo, Dan Zhang, Xiangbo Xu, Fernando Gomes Romeiro, Andrea Mancuso, Jingqiao Zhang, Ruirui Feng, Xinmiao Zhou, Cen Hong, Xingshun Qi

**Affiliations:** ^1^Liver Cirrhosis Group, Department of Gastroenterology, General Hospital of Northern Theater Command (Formerly General Hospital of Shenyang Military Area), Shenyang, China; ^2^Department of Clinical Laboratory, The First Hospital of Lanzhou University, Lanzhou, China; ^3^Department of General Surgery, General Hospital of Northern Theater Command (Formerly General Hospital of Shenyang Military Area), Shenyang, China; ^4^Department of Pharmaceutical Sciences, Shenyang Pharmaceutical University, Shenyang, China; ^5^Faculdade de Medicina de Botucatu UNESP, Campus de Botucatu, S/N, Botucatu, SP, Brazil; ^6^Department of Internal Medicine, ARNAS Civico, Palermo, Italy

## Abstract

**Methods:**

Cirrhotic patients who were consecutively hospitalized between January 2016 and March 2019 were screened. Serum cardiac biomarkers at admission, including N-Terminal pro-B-type natriuretic peptide (NT-pro BNP), high-sensitivity cardiac troponin T (hs-cTnT), creatine kinase (CK), creatine kinase MB (CK-MB), and lactate dehydrogenase (LDH), were collected. Acute decompensating events at admission, primarily including ascites, acute gastrointestinal hemorrhage, and acute-on-chronic liver failure (ACLF), were recorded.

**Results:**

The NT-pro BNP level was significantly higher in cirrhotic patients with acute decompensating events than in those without any decompensating events (median: 140.75 pg/mL versus 41.86 pg/mL, *P* < 0.001). The NT-pro BNP level significantly correlated with ascites, acute gastrointestinal hemorrhage, and ACLF. The hs-cTnT level was significantly higher in cirrhotic patients with acute decompensating events than in those without decompensating events (median: 0.008 ng/mL versus 0.006 ng/mL, *P* = 0.007). The hs-cTnT level significantly correlated with acute gastrointestinal hemorrhage, but not ascites or ACLF. LDH (185.0 U/L versus 173.5 U/L, *P* = 0.281), CK (71 U/L versus 84 U/L, *P* = 0.157), and CK-MB (29.5 U/L versus 33.0 U/L, *P* = 0.604) levels were not significantly different between cirrhotic patients with and without acute decompensating events.

**Conclusion:**

The elevated NT-pro BNP level seems to be closely related to the development of acute decompensating events in liver cirrhosis.

## 1. Introduction

Liver cirrhosis is a state of systemic hyperdynamic circulation characterized by increased cardiac output and decreased peripheral resistance, especially in the presence of decompensating events [1]. This disease activates the sympathetic nervous system (SNS) and the renin-angiotensin-aldosterone system (RAAS), which further increases the myocardial tension and ultimately results in chronic cardiac dysfunction [2–4]. Consequently, the levels of atrial natriuretic peptide (ANP), brain natriuretic peptide (BNP), and troponin I (TnI) are increased [5]. Such chronic cardiac dysfunction in liver cirrhosis presenting with systolic dysfunction, diastolic dysfunction, and electrophysiological changes is known as “cirrhotic cardiomyopathy” [4]. An interaction between liver cirrhosis and cardiac dysfunction suggests that serum cardiac markers may be valuable in evaluating the disease state of liver cirrhosis.

BNP and N-Terminal pro-B-type natriuretic peptide (NT-pro BNP) are secreted in response to increased myocardial stress [6–8]. The NT-pro BNP level is significantly increased in liver cirrhosis, which may be related to cardiac dysfunction [9–11]. Also, the NT-pro BNP level significantly correlates with the severity of liver dysfunction and prognosis of cirrhotic patients [7, 12]. On the other hand, high-sensitivity cardiac troponin T (hs-cTnT), another highly specific and sensitive marker of myocardial injury, is also significantly increased in patients with liver cirrhosis. Thus, NT-pro BNP as well as hs-cTnT may be valuable for prognostic assessment of liver cirrhosis [12]. However, their correlation with acute decompensating events in liver cirrhosis has never been explored yet. Additionally, the clinical significance of other biomarkers of cardiac injury, such as creatine kinase (CK), creatine kinase MB (CK-MB), and lactate dehydrogenase (LDH), in liver cirrhosis remains unclear.

Therefore, this study is aimed at exploring the relationship between these cardiac markers and decompensating events in cirrhosis.

## 2. Methods

### 2.1. Patients

We have prospectively collected the demographic, clinical, and laboratory data of cirrhotic patients who were consecutively admitted to the Department of Gastroenterology of the General Hospital of Northern Theater Command (formerly General Hospital of Shenyang Military Area) and treated by an attending physician (XQ) since January 1, 2016. Until March 31, 2019, there were a total of 761 admissions. We retrospectively screened the patients who had undergone the evaluation of laboratory data regarding serum cardiac markers during the hospitalizations. Exclusion criteria were as follows: (1) confirmed or suspected diagnosis of malignancy, (2) severe renal insufficiency (estimated glomerular filtration rate (eGFR) < 30 mL/min/1.73 m^2^), (3) cardiac diseases (i.e., heart failure, coronary atherosclerotic heart disease, and atrial fibrillation), (4) ischemic stroke, and (5) absence of data regarding serum cardiac markers detected during the hospitalizations. Repeated admissions of the same patient were not deliberately excluded, because this study focused on the in-hospital outcome and decompensating events during the hospitalizations, but not on long-term follow-up outcomes. If a patient had multiple measurements of serum cardiac markers during the same hospitalization, we selected the data obtained at the first time of blood collection. The study protocol was approved by the Medical Ethics Committee of our hospital. We primarily collected demographic data, etiology of liver cirrhosis, decompensating events at admissions, and laboratory tests including serum cardiac markers.

### 2.2. Definitions and Diagnosis

Acute gastrointestinal hemorrhage was defined as previously described [13, 14]. The Child-Pugh score was calculated [15]. Model for end-stage liver disease (MELD) and MELD with sodium (MELD-Na) scores were calculated according to an equation updated by the OPTN/UNOS (American Organ Acquisition and Transplantation Network/Organ Resource Sharing Network) in 2016 [16], as follows:

(1) $$\begin{array}{c} MELD\;\left(i\right)=9.57\times \ln\;\left(\text{creatinine mg}/\text{dL}\right)+3.78\times \ln\;\left(\text{bilirubin mg}/\text{dL}\right)+11.2\times \ln\;\left(\text{INR}\right)+6.43,\\ MELD‐Na=MELD\;\left(i\right)+1.32\times \left(137‐Na\right)-\left[0.033\times MELD\;\left(i\right)\times \left(137‐Na\right)\right.. \end{array}$$

If Na^+^ < 125 mmol/L, it is set to 125; if Na^+^ > 137, it is set to 137.

The grade of ascites was defined according to the consensus of the International Ascites Club [17]. Patients with acute-on-chronic liver failure (ACLF) were identified by the recommendations of the Asian Pacific Association for the Study of the Liver (APASL) consensus [18, 19]. Severe renal insufficiency was defined as eGFR < 30 mL/min/1.73 m^2^ [20]. The eGFR was calculated using the simplified equation [21], as follows:

(2) $$\begin{array}{c} e\text{GFR }\left(\text{mL}/\min\text{ per }1.73\;m^{2}\right)=186.3\times \text{serum creatinine concentration }\left(\text{mg}/\text{dL}\right)\;\left(\exp\;\left[-1.154\right]\right)\times age\;\left(\exp\;\left[-0.203\right]\right)\times \left(0.742\;\text{if }female\right)\times \left(1.212\;\text{if }black\right). \end{array}$$

### 2.3. Groups

We divided cirrhotic patients into 5 groups: (1) cirrhotic patients without acute decompensating events, (2) cirrhotic patients with acute decompensating events, (3) cirrhotic patients with ascites, (4) cirrhotic patients with acute gastrointestinal hemorrhage, and (5) cirrhotic patients with ACLF.

### 2.4. Measurement of Serum Cardiac Markers

All serum cardiac markers were measured at the Department of Laboratory of our hospital. They included NT-pro BNP detected by enzyme-linked immunosorbent assay (ELISA) (double antibody sandwich method) with a normal range of 0-125 pg/mL, hs-cTnT by ELISA (double antibody sandwich method) with a normal range of 0-0.05 ng/mL, CK by coupled-enzyme assay with a normal range of 38-174 U/L, CK-MB by immune inhibition assay with a normal range of 0-24 U/L, LDH by the spectrophotometric method with a normal range of 109-245 U/L, and hs-CRP by latex immune turbidimetry with a normal range of 0-3 mg/L. Only the data obtained at the first time of measurement were selected, thus avoiding the influence of drugs used during hospitalization.

### 2.5. Statistical Analyses

Continuous data were expressed as mean ± standard deviation and median (quartiles) and were compared by using the Wilcoxon rank-sum test. Categorical data were expressed as frequency (percentage) and were compared by using the chi-square test. Considering that age and gender are important factors influencing serum NT-pro BNP and hs-cTnT levels [22–26], partial correlation analysis was adjusted for age and gender to analyze the correlation of serum NT-pro BNP and hs-cTnT with liver disease conditions. Pearson or Spearman tests were performed to analyze the correlation between disease conditions and other serological cardiac markers, such as CK, CK-MB, and LDH. Multivariate linear regression analysis was performed to analyze the correlation of serological cardiac markers with categorical variables. A two-sided *P* < 0.05 was considered to be statistically significant. SPSS statistics software version R23.0.0.0 was employed to perform all statistical analyses.

## 3. Results

### 3.1. Patients

**Figure 1:**
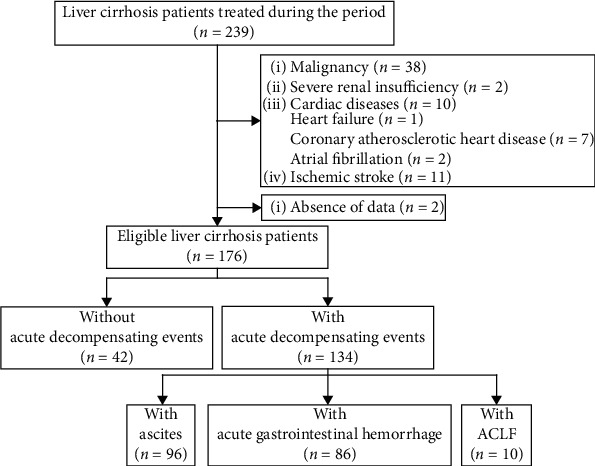
A flow chart of patient selection.

Overall, 176 patients with liver cirrhosis were included (Figure 1), of whom 42 (23.86%) did not have any decompensating events but conducted regular follow-up and/or prophylactic endoscopic variceal treatment and 134 (76.14%) had acute decompensating events, including ascites (*n* = 96, 71.64%), acute gastrointestinal hemorrhage (*n* = 86, 64.18%), and ACLF (*n* = 10, 7.46%).

### 3.2. Comparison between Cirrhotic Patients with and without Decompensating Events

**Figure 2:**
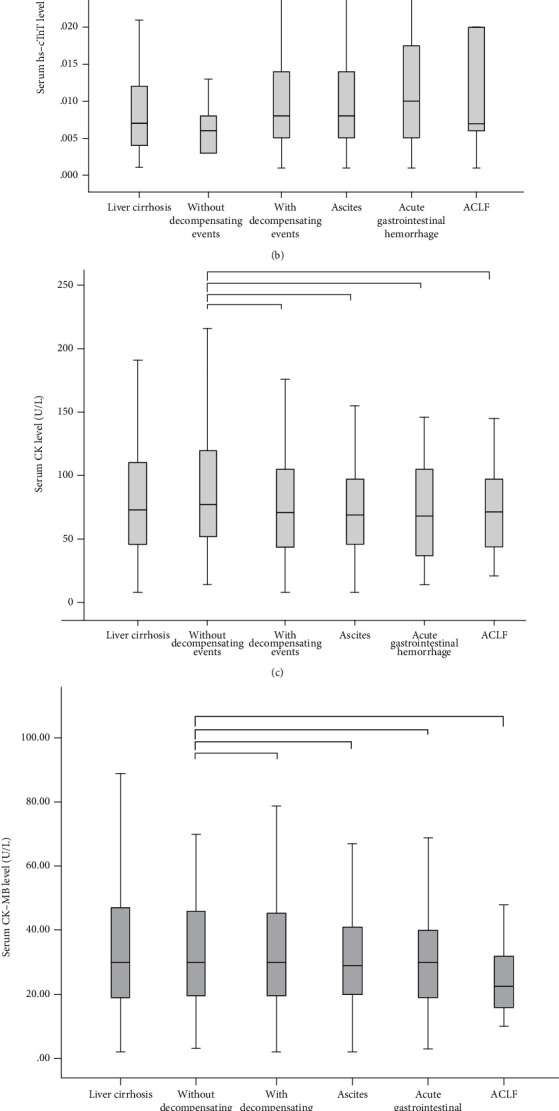
Box plots showing the concentrations of serum NT-pro BNP (a), hs-cTnT (b), CK (c), CK-MB (d), and LDH (e) in general cirrhotic patients, cirrhotic patients with and without decompensating events, cirrhotic patients with ascites, cirrhotic patients with acute gastrointestinal hemorrhage, and cirrhotic patients with ACLF. Notes: ^∗∗^*P* < 0.001 and ^∗^*P* < 0.05.

Cirrhotic patients with decompensating events had significantly higher levels of NT-pro BNP (*P* < 0.001) and hs-cTnT (*P* = 0.007) than those without decompensating events (Figure 2), but the differences in the levels of CK, CK-MB, and LDH were not significant between them (Table 1).

Cirrhotic patients with ascites had significantly higher levels of NT-pro BNP (*P* < 0.001) and hs-cTnT (*P* = 0.002) than those without decompensating events (Figure 2), but the differences in the levels of CK, CK-MB, and LDH were not significant between them (Table 2).

Cirrhotic patients with acute gastrointestinal hemorrhage had significantly higher levels of NT-pro BNP (*P* < 0.001) and hs-cTnT (*P* = 0.003) than those without decompensating events (Figure 2), but the differences in the levels of CK, CK-MB, and LDH were not significant between them (Table 2).

Cirrhotic patients with ACLF had a significantly higher level of NT-pro BNP (*P* < 0.001) than those without decompensating events (Figure 2), but the differences in the levels of CK, CK-MB, and LDH were not significant between them (Table 2).

### 3.3. Correlation of Serum Cardiac Markers with Child-Pugh and MELD Scores in Patients with Liver Cirrhosis

Partial correlation analyses demonstrated that the NT-pro BNP level significantly correlated with Child-Pugh and MELD scores. These correlations were observed in all the cirrhotic patients and in those with decompensating events, but not in those without decompensating events (Table 3). Partial correlation analyses demonstrated that the hs-cTnT level had no significant correlation with Child-Pugh and MELD scores in cirrhotic patients regardless of the presence of decompensating events (Supplementary Table 1). Correlation analyses demonstrated that CK (Supplementary Table 2) and CK-MB (Supplementary Table 3) levels did not significantly correlate with Child-Pugh and MELD scores in cirrhotic patients. On the other hand, the LDH level significantly correlated with Child-Pugh and MELD scores in cirrhotic patients (Supplementary Table 4).

### 3.4. Correlation between Serum Cardiac Markers and Decompensating Events in Cirrhotic Patients

Age- or gender-adjusted multivariate linear regression analyses demonstrated that the NT-pro BNP level significantly correlated with overall acute decompensating events, ascites, acute gastrointestinal hemorrhage, and ACLF (Table 4); the hs-cTnT level significantly correlated with overall acute decompensating events and acute gastrointestinal hemorrhage, but not ascites or ACLF (Supplementary Table 5); CK and CK-MB levels did not significantly correlate with overall acute decompensating events, ascites, acute gastrointestinal hemorrhage, or ACLF; the LDH level significantly correlated with ascites, acute gastrointestinal hemorrhage, and ACLF, but not overall acute decompensating events (Supplementary Table 6).

## 4. Discussion

In this retrospective observational study, we rigorously screened the participants by excluding the confounding factors, which makes our statistical results more reliable. Additionally, we included a relatively large number of cirrhotic patients, which makes our conclusions more representative. The major findings are as follows: (i) the NT-pro BNP level was significantly higher in decompensated cirrhosis. (ii) The NT-pro BNP level also significantly correlated with Child-Pugh and MELD scores in cirrhosis with acute decompensation, but not in those without decompensation. (iii) The hs-cTnT level was elevated in cirrhosis with acute decompensation but was unrelated to the liver disease severity. (iv) The LDH level significantly correlated with Child-Pugh and MELD scores in cirrhosis, but was unrelated to decompensating events. (v) CK and CK-MB levels were neither significantly increased in cirrhosis with decompensation nor correlated with Child-Pugh and MELD scores.

### 4.1. NT-pro BNP

NT-pro BNP, a prohormone of BNP, is secreted into the systemic circulation by cardiac ventricles in response to myocardial hypertrophy and is involved in the regulation of cardiac volume homeostasis [27–29]. Thus, the NT-pro BNP level is often considered as an effective and useful marker for screening of early stages of cardiac dysfunction [30]. As we know, cirrhotic cardiomyopathy is a chronic cardiac systolic and diastolic dysfunction in cirrhotic patients in the absence of prior heart disease [31, 32]. There is no obvious abnormal change of cardiac function in the resting state; besides, a decreased afterload in cirrhosis often results in normal or even increased left ventricular ejection fraction [31]. Thus, noninvasive cardiac biomarkers are potentially useful to reflect the slight change of pressure state of end-diastolic wall stress and intracardiac filling pressures.

The serum NT-pro BNP level is significantly higher in patients with liver cirrhosis [7, 9, 10, 12, 33], probably because it is often associated with hyperdynamic circulation, such as increased heart rate and cardiac output, thereby impairing cardiac contractility [34–36]. Our study for the first time found that the serum NT-pro BNP level was significantly higher in cirrhotic patients who suffer an acute decompensation, such as ascites, gastrointestinal hemorrhage, and ACLF, when the values were compared to those without decompensation. Interestingly, we also found that the NT-pro BNP level significantly correlated with Child-Pugh and MELD scores in cirrhotic patients with acute decompensating events, but not in those without decompensation, which would suggest that NT-pro BNP can reflect the insidious change of cardiac dysfunction in advanced cirrhosis with cardiac dysfunction.

In addition, BNP is a natriuretic hormone released from myocardial cells in response to volume expansion, end-diastolic wall stress, and possibly increased intracardiac filling pressures [8, 37]. Hypertrophy of the left ventricle, left-atrial dilatation, and increased end-diastolic and end-systolic left-ventricular volume are frequently observed in liver cirrhosis [1, 32, 38, 39], which are potentially the main causes for an increase of NT-pro BNP.

### 4.2. hs-cTnT

hs-cTnT, a protein complex regulating the contraction of striated muscle, is released when myocardial ischemia induces nonreversible injury of myocardial tissue [40]. hs-cTnT is a specific and sensitive biomarker of myocardial damage and is being widely used for clinical screening in patients with suspected acute myocardial infarction [41, 42], but not for evaluating the change of myocardial contractility. The hs-cTnT level can be also elevated in some cardiac and noncardiac conditions, such as severe renal insufficiency [43–46], tachycardia, pericarditis, vigorous exercise [47], and atrial fibrillation [48–50]. The present study has rigorously excluded these conditions. The hs-cTnT level seems to be related to the severity and survival of cirrhotic patients [12, 14]. Our results also showed that the hs-cTnT level was significantly higher in decompensated cirrhotic patients than those without decompensation. This association was mainly attributed to the effect of acute gastrointestinal hemorrhage, but not to ascites or ACLF (Supplementary Table 5). A possible explanation for this finding could be that acute gastrointestinal bleeding in cirrhotic patients may lead to hypovolemic hypotension, which is a significant risk factor for myocardial damage [51–53], thereby increasing the levels of myocardial damage biomarkers [54]. The pathophysiological link of the association remains unexplained, and it needs further research to clarify its mechanism.

### 4.3. CK and CK-MB

We did not find any significant difference in CK and CK-MB levels, comparing compensated and decompensated cirrhotic patients. Moreover, there was no correlation of CK and CK-MB levels with Child-Pugh and MELD scores in cirrhosis. These analyses were performed in the groups with and without decompensating events. Traditionally, CK-MB is helpful for estimating the infarct size in acute myocardial infarction and is highly specific to heart tissue [55, 56] while CK is used for assessing myocardial damage in acute myocardial infarction [57]. None of them is a good indicator of cardiac volume overload. Our study suggested that CK and CK-MB levels did not correlate with the severity of cirrhosis.

### 4.4. LDH

LDH, a cytoplasmic enzyme, exists in a wide range of tissues and is elevated when cells are damaged. LDH is not specific for the diagnosis of a disease. There are five types of serum LDH isoenzymes. Among them, LDH1 is mainly derived from the heart and LDH5 from the liver [58]. However, LDH5 have lower specificity and sensitivity than ALT for diagnosing and evaluating liver diseases [59]. LDH significantly correlated with Child-Pugh and MELD scores in cirrhotic patients. However, there was no significant difference in LDH levels between cirrhotic patients with and without decompensation. These results indicate that LDH might not be sensitive to early cardiac dysfunction caused by cirrhosis.

### 4.5. Limitations

First, the number of patients with ACLF was small in our cohort and the relationship between serum cardiac markers and ACLF needs further clarification. Second, a reasonable and convenient approach for quantifying blood loss volume during acute gastrointestinal hemorrhage and volume of ascites was unavailable. Third, healthy controls may make the results more comprehensive. Further studies should further consider the effects of the severity of such decompensating events on the long-term prognosis.

## 5. Conclusion

An elevated NT-pro BNP level might be useful to identify the cardiac volume overload caused by acute decompensating events in advanced cirrhosis. Additionally, the hs-cTnT level was elevated in cirrhosis with acute decompensating events.

## Figures and Tables

**Table 1:** Comparison of characteristics of cirrhotic patients without and with decompensating events.

Variables	Without decompensation		With decompensation		*P* value
	No. Pts	Mean ± SD; median (range) or frequency (percentage)	No. Pts	Mean ± SD; median (range) or frequency (percentage)	
Sex (male/female), *n* (%)	42	35 (83.33%)/7 (16.67%)	134	89 (66.42%)/45 (33.58%)	0.051
Age (years)	42	52.74 ± 11.03; 52.5 (46.50-62.25)	134	56.36 ± 12.16; 55.00 (47.00-65.00)	0.130
Causes of liver diseases, *n* (%)	42		134		
Hepatitis B virus alone	14	14 (33.33%)	48	48 (35.82%)	/
Hepatitis C virus alone	4	4 (9.52%)	9	9 (6.72%)	/
Alcohol alone	12	12 (28.57%)	29	29 (21.64%)	/
Hepatitis B virus+alcohol	1	1 (2.38%)	4	4 (2.99%)	/
Autoimmune	1	1 (2.38%)	16	16 (11.94%)	/
Drug	1	1 (2.38%)	6	6 (4.48%)	/
Others	1	1 (2.38%)	0	0 (0%)	/
Red blood cell (10^12^/L)	42	3.99 ± 0.64; 4.05 (3.62-4.57)	134	3.16 ± 0.79; 3.10 (2.56-3.76)	**<0.001**
Hemoglobin (g/L)	42	117.07 ± 23.75; 123.00 (108.00-131.25)	134	93.48 ± 27.35; 91.50 (70.00-115.00)	**<0.001**
Hematocrit (%)	42	35.65 ± 6.58; 36.60 (32.88-39.63)	134	28.49 ± 7.88; 28.20 (21.90-34.73)	**<0.001**
White blood cell (10^9^/L)	42	3.83 ± 1.97; 3.35 (2.45-4.65)	134	4.71 ± 3.14; 3.70 (2.60-6.03)	0.155
Platelet count (10^9^/L)	42	87.67 ± 57.48; 71.00 (48.50-104.75)	134	97.04 ± 74.17; 76.00 (53.50-107.50)	0.433
Total bilirubin (*μ*mol/L)	42	32.22 ± 34.77; 22.90 (16.15-32.45)	134	43.84 ± 60.88; 23.15 (15.15-45.13)	0.682
Direct bilirubin (*μ*mol/L)	42	16.32 ± 24.77; 9.90 (6.25-13.75)	134	25.94 ± 45.74; 10.50 (6.08-20.73)	0.351
Alanine aminotransferase (U/L)	42	68.38 ± 216.68; 26.29 (17.86-37.58)	134	31.96 ± 30.04; 23.43 (15.80-38.64)	0.391
Aspartate aminotransferase (U/L)	42	62.81 ± 130.73; 32.73 (24.25-50.47)	134	49.40 ± 44.79; 32.71 (23.91-59.55)	0.753
Alkaline phosphatase (U/L)	42	126.48 ± 121.34; 96.34 (77.34-139.58)	134	121.54 ± 97.12; 95.93 (67.99-143.13)	0.612
Gamma-glutamyl transpeptidase (U/L)	42	120.56 ± 274.49; 49.94 (27.53-83.88)	134	91.17 ± 154.74; 41.56 (19.87-93.42)	0.429
Albumin (g/L)	42	36.69 ± 5.40; 37.35 (32.38-41.03)	134	30.87 ± 6.58; 30.80 (25.60-35.80)	**<0.001**
Blood urea nitrogen (mmol/L)	42	5.15 ± 1.75; 4.87 (3.79-6.19)	134	6.69 ± 3.49; 6.05 (4.39-7.87)	**0.007**
Creatinine (*μ*mol/L)	42	65.47 ± 14.14; 64.68 (56.02-74.81)	134	69.58 ± 23.03; 65.20 (53.53-78.15)	0.694
eGFR (mL/min/1.73 m^2^)	42	119.18 ± 30.56; 118.40 (97.75-133.97)	134	109.63 ± 36.25; 106.52 (84.22-133.52)	0.068
Na (mmol/L)	42	139.02 ± 2.52; 139.45 (137.80-140.43)	134	138.10 ± 4.67; 138.45 (136.00-141.00)	0.226
Prothrombin time (second)	42	16.22 ± 2.05; 16.05 (15.03-16.88)	134	17.48 ± 3.47; 16.70 (15.10-18.90)	**0.033**
Activated partial thromboplastin time (second)	42	42.41 ± 5.47; 41.60 (38.98-45.53)	134	41.68 ± 7.40; 40.15 (37.10-44.30)	0.149
International normalized ratio	42	1.31 ± 0.21; 1.28 (1.19-1.38)	134	1.44 ± 0.37; 1.36 (1.20-1.58)	**0.040**
D-dimer (mg/L)	41	1.19 ± 1.68; 0.60 (0.29-1.27)	131	3.07 ± 3.73; 1.81 (0.72-4.41)	**<0.001**
High-sensitivity C-reactive protein (mg/L)	41	5.39 ± 8.05; 2.20 (0.90-6.60)	133	15.25 ± 27.70; 5.70 (1.35-14.10)	**0.016**
MELD score	42	11.20 ± 3.10; 10.26 (9.04-13.06)	134	13.40 ± 5.75; 11.32 (9.22-16.56)	0.093
Child-Pugh score	42	5.90 ± 1.10; 6.00 (5.00-6.00)	134	7.82 ± 2.18; 7.00 (6.00-9.00)	**<0.001**
Child-Pugh class	42		134		
A, *n* (%)	33	33 (78.57%)	44	44 (32.84%)	**<0.001**
B, *n* (%)	9	9 (21.43%)	58	58 (43.28%)	**<0.001**
C, *n* (%)	0	0 (0%)	32	32 (23.88%)	**<0.001**
NT-pro BNP (pg/mL)	38	58.20 ± 56.57; 41.86 (20.36-69.16)	120	402.32 ± 1013.60; 140.75 (62.34-401.33)	**<0.001**
High-sensitivity cardiac troponin T (ng/mL)	42	0.007 ± 0.005; 0.006 (0.003-0.008)	128	0.017 ± 0.045; 0.008 (0.015-0.003)	**0.007**
Creatine kinase (U/L)	42	93.93 ± 50.81; 84.00 (53.50-123.75)	132	98.88 ± 103.56; 71.00 (44.00-105.50)	0.157
Creatine kinase MB (U/L)	42	35.90 ± 21.51; 33.00 (19.00-47.25)	132	33.62 ± 18.93; 29.5 (19.25-46.25)	0.604
Lactate dehydrogenase (U/L)	42	184.00 ± 55.87; 173.50 (137.50-211.50)	132	194.17 ± 58.30; 185.00 (152.00-226.75)	0.281

Bold font indicates statistically significant *P* values. Abbreviations: eGFR: the estimated glomerular filtration rate; MELD: model for end-stage liver disease; NT-pro BNP: N-Terminal pro-B-type natriuretic peptide.

**Table 2:** Comparison of characteristics of cirrhotic patients with ascites, acute gastrointestinal hemorrhage, ACLF, and without decompensating events.

Variables	With ascites			With acute gastrointestinal hemorrhage			With ACLF		
	No. Pts	Mean ± SD; median (range) or frequency (percentage)	*P* value	No. Pts	Mean ± SD; median (range) or frequency (percentage)	*P* value	No. Pts	Mean ± SD; median (range) or frequency (percentage)	*P* value
Sex (male/female), *n* (%)	96	64 (66.67%)/32 (33.33%)	0.064	86	56 (65.12%)/30 (34.88%)	**0.039**	10	8 (80%)/2 (20%)	0.558
Age (years)	96	58.17 ± 11.88; 60.00 (48.00-65.75)	**0.027**	86	53.72 ± 11.37; 54.00 (45.00-63.25)	0.692	10	53.20 ± 14.85; 48.00 (45.00-57.00)	0.429
Red blood cell (10^12^/L)	96	3.15 ± 0.77; 3.08 (2.57-3.83)	**<0.001**	86	2.95 ± 0.76; 2.75 (2.39-3.35)	**<0.001**	10	2.59 ± 0.85; 2.60 (1.78-3.45)	**<0.001**
Hemoglobin (g/L)	96	93.27 ± 27.38; 93.00 (70.00-113.75)	**<0.001**	86	83.74 ± 25.01; 76.00 (66.00-99.00)	**<0.001**	10	92.60 ± 28.50; 98.50 (65.25-117.25)	0.018
Hematocrit (%)	96	28.41 ± 7.78; 28.40 (21.38-34.33)	**<0.001**	86	25.69 ± 7.34; 23.15 (20.33-30.20)	**<0.001**	10	27.09 ± 8.27; 28.80 (18.58-34.75)	0.004
White blood cell (10^9^/L)	96	4.89 ± 3.41; 3.85 (2.73-6.18)	0.119	86	4.80 ± 3.57; 3.60 (2.38-6.30)	0.390	10	6.21 ± 3.02; 6.15 (4.43-8.25)	0.021
Platelet count (10^9^/L)	96	98.94 ± 78.63; 78.00 (54.00-106.75)	0.384	86	96.03 ± 77.77; 72.50 (51.00-103.75)	0.755	10	73.60 ± 45.68; 64.50 (39.00-97.25)	0.562
Total bilirubin (*μ*mol/L)	96	52.38 ± 69.82; 28.90 (15.38-63.15)	0.186	86	33.63 ± 47.34; 19.55 (13.20-32.20)	0.292	10	199.84 ± 121.74; 166.20 (121.25-223.50)	**<0.001**
Direct bilirubin (*μ*mol/L)	96	32.73 ± 52.48; 13.45 (6.43-37.53)	**0.039**	86	19.02 ± 37.59; 8.45 (5.40-16.23)	0.582	10	141.24 ± 97.61; 105.10 (82.08-184.23)	**<0.001**
Alanine aminotransferase (U/L)	96	33.89 ± 33.87; 23.09 (16.25-39.37)	0.517	86	29.02 ± 26.62; 20.92 (14.62-36.63)	0.132	10	64.24 ± 61.31; 51.43 (23.77-63.76)	0.012
Aspartate aminotransferase (U/L)	96	55.35 ± 50.57; 34.56 (24.01-68.95)	0.404	86	41.78 ± 40.40; 27.45 (20.43-48.41)	0.232	10	109.25 ± 70.69; 93.55 (65.65-128.31)	**<0.001**
Alkaline phosphatase (U/L)	96	132.01 ± 106.30; 101.99 (75.12-149.67)	0.621	86	98.19 ± 61.17; 81.09 (59.99-110.37)	**0.019**	10	132.94 ± 51.66; 137.08 (84.14-169.98)	0.202
Gamma-glutamyl transpeptidase (U/L)	96	94.49 ± 133.18; 46.21 (21.10-108.21)	0.901	86	70.20 ± 144.14; 29.01 (16.74-57.98)	**0.032**	10	75.11 ± 44.44; 67.05 (37.05-97.91)	0.218
Albumin (g/L)	96	29.70 ± 6.34; 29.65 (25.10-33.40)	**<0.001**	86	31.31 ± 6.18; 31.10 (25.68-36.18)	**<0.001**	10	23.52 ± 4.40; 22.80 (19.95-26.98)	**<0.001**
Blood urea nitrogen (mmol/L)	96	6.78 ± 3.64; 6.05 (4.42-7.86)	**0.007**	86	7.18 ± 3.95; 6.25 (4.39-8.46)	**0.003**	10	8.13 ± 5.06; 6.26 (4.78-11.15)	0.070
Creatinine (*μ*mol/L)	96	71.20 ± 25.69; 63.55 (53.23-82.68)	0.686	86	68.14 ± 22.75; 65.86 (52.78-75.94)	0.895	10	74.36 ± 28.63; 61.15 (52.53-101.95)	0.763
eGFR (mL/min/1.73 m^2^)	96	108.05 ± 38.74; 104.38 (77.60-133.84)	**0.047**	86	112.93 ± 37.30; 108.07 (86.83-138.32)	0.247	10	110.64 ± 43.03; 107.84 (73.24-137.83)	0.403
Na (mmol/L)	96	137.54 ± 5.13; 137.75 (134.93-140.95)	**0.041**	86	138.66 ± 4.80; 139.35 (136.55-141.03)	0.741	10	135.05 ± 5.57; 135.20 (131.55-139.15)	0.021
Prothrombin time (second)	96	18.17 ± 3.74; 17.15 (15.60-20.45)	**0.001**	86	17.69 ± 3.55; 16.90 (15.20-19.10)	**0.026**	10	24.30 ± 4.63; 22.85 (20.65-27.95)	**<0.001**
Activated partial thromboplastin time (second)	96	43.11 ± 7.89; 41.90 (37.73-47.28)	0.987	86	41.37 ± 7.12; 39.70 (36.98-43.98)	0.064	10	52.95 ± 8.64; 52.85 (46.63-55.95)	**<0.001**
International normalized ratio	96	1.52 ± 0.40; 1.40 (1.25-1.75)	**0.001**	86	1.47 ± 0.38; 1.37 (1.21-1.60)	**0.031**	10	2.18 ± 0.53; 1.99 (1.77-2.59)	**<0.001**
D-dimer (mg/L)	95	3.53 ± 4.04; 2.28 (0.93-4.64)	**<0.001**	83	2.31 ± 2.30; 1.43 (0.58-3.57)	**0.001**	9	7.79 ± 8.54; 4.51 (3.41-9.02)	**<0.001**
High-sensitivity C-reactive protein (mg/L)	95	18.93 ± 31.44; 6.90 (2.20-20.30)	**0.001**	85	13.88 ± 26.58; 4.50 (1.20-11.55)	0.071	10	44.50 ± 36.88; 30.65 (11.35-75.35)	**<0.001**
MELD score	96	14.79 ± 6.10; 13.50 (10.07-18.23)	**0.001**	86	12.68 ± 5.29; 10.65 (9.13-14.42)	0.343	10	25.81 ± 4.68; 28.14 (21.24-30.04)	**<0.001**
Child-Pugh score	96	8.57 ± 2.04; 8.00 (7.00-10.00)	**<0.001**	86	7.29 ± 2.07; 7.00 (6.00-8.00)	**<0.001**	10	12.10 ± 1.10; 12.00 (11.75-13.00)	**<0.001**
NT-pro BNP (pg/mL)	87	458.09 ± 1167.73; 165.20 (71.06-415.80)	**<0.001**	78	459.77 ± 1208.86; 144.95 (60.82-443.93)	**<0.001**	10	1511.39 ± 3188.84; 212.30 (150.70-1662.75)	**<0.001**
High-sensitivity cardiac troponin T (ng/mL)	92	0.015 ± 0.018; 0.008 (0.005-0.014)	**0.002**	81	0.020 ± 0.055; 0.009 (0.005-0.014)	**0.003**	9	0.024 ± 0.033; 0.007 (0.005-0.035)	0.157
Creatine kinase (U/L)	95	90.65 ± 83.30; 69.00 (45.00-97.00)	0.099	84	101.63 ± 117.93; 69.00 (40.50-105.50)	0.117	10	99.30 ± 98.38; 71.50 (41.25-109.00)	0.501
Creatine kinase MB (U/L)	95	32.72 ± 18.73; 29.00 (20.00-42.00)	0.463	84	33.13 ± 18.40; 29.50 (19.00-43.75)	0.552	10	29.60 ± 24.40; 22.50 (14.50-36.00)	0.163
Lactate dehydrogenase (U/L)	95	201.85 ± 57.99; 195.00 (154.00-232.00)	0.076	84	181.51 ± 58.43; 171.00 (140.00-206.25)	0.727	10	238.30 ± 80.65; 235.00 (157.50-317.25)	0.050

Bold font indicates statistically significant *P* values. Abbreviations: ACLF: acute-on-chronic liver failure; eGFR: the estimated glomerular filtration rate; MELD: model for end-stage liver disease; NT-pro BNP: N-Terminal pro-B-type natriuretic peptide.

**Table 3:** Partial correlation analysis of the NT-pro BNP level in cirrhosis.

Variables	All liver cirrhosis			Liver cirrhosis without decompensation			Liver cirrhosis with decompensation		
	No. Pts	*P* value	Correlation coefficient	No. Pts	*P* value	Correlation coefficient	No. Pts	*P* value	Correlation coefficient
Age (years)	/	Controlling	/	/	Controlling	/	/	Controlling	/
Sex (male/female), *n* (%)	/	Controlling	/	/	Controlling	/	/	Controlling	/
Red blood cell (10^12^/L)	154	**<0.001**	-0.322	34	**0.023**	-0.379	116	**0.001**	-0.313
Hemoglobin (g/L)	154	**0.004**	-0.229	34	**0.001**	-0.540	116	**0.025**	-0.207
Hematocrit (%)	154	**0.001**	-0.254	34	**0.001**	-0.523	116	**0.011**	-0.234
White blood cell (10^9^/L)	154	**0.027**	0.177	34	0.093	-0.284	116	0.058	0.175
Platelet count (10^9^/L)	154	0.894	-0.011	34	0.201	-0.218	116	0.842	-0.018
Total bilirubin (*μ*mol/L)	154	**0.027**	0.178	34	0.755	-0.054	116	0.054	0.178
Direct bilirubin (*μ*mol/L)	154	0.063	0.149	34	0.661	-0.076	116	0.113	0.146
Alanine aminotransferase (U/L)	154	0.715	-0.029	34	0.534	-0.107	116	0.609	-0.048
Aspartate aminotransferase (U/L)	154	0.694	-0.032	34	0.493	-0.118	116	0.687	-0.037
Alkaline phosphatase (U/L)	154	0.534	-0.050	34	0.071	-0.304	116	0.623	-0.046
Gamma-glutamyl transpeptidase (U/L)	154	0.983	-0.002	34	0.206	-0.216	116	0.794	0.024
Albumin (g/L)	154	**0.006**	-0.219	34	0.263	-0.192	116	**0.042**	-0.188
Blood urea nitrogen (mmol/L)	154	**0.022**	0.183	34	0.099	0.279	116	0.074	0.165
Creatinine (*μ*mol/L)	154	0.692	-0.032	34	0.755	-0.054	116	0.547	-0.056
eGFR (mL/min/1.73 m^2^)	154	0.073	0.144	34	0.823	0.039	116	0.058	0.175
Sodium (mmol/L)	154	**0.039**	-0.165	34	0.586	0.094	116	0.082	-0.161
Prothrombin time (second)	154	**<0.001**	0.416	34	0.480	0.122	116	**<0.001**	0.419
Activated partial thromboplastin time (second)	154	**0.023**	0.182	34	0.406	0.143	116	**0.030**	0.200
International normalized ratio	154	**<0.001**	0.436	34	0.380	0.151	116	**<0.001**	0.439
D-dimer (mg/L)	153	**0.003**	0.241	34	0.125	0.260	115	**0.040**	0.190
High-sensitivity C-reactive protein (mg/L)	153	**<0.001**	0.285	33	0.576	0.098	116	**0.003**	0.270
MELD score	154	**<0.001**	0.302	34	0.701	0.066	116	**0.001**	0.296
Child-Pugh score	154	**<0.001**	0.346	34	0.279	0.185	116	**<0.001**	0.325
High-sensitivity cardiac troponin T (ng/mL)	151	**0.004**	0.229	34	0.062	-0.315	113	**0.026**	0.208
Creatine kinase (U/L)	154	0.653	-0.036	34	0.567	0.099	116	0.588	-0.050
Creatine kinase MB (U/L)	154	0.209	-0.101	34	0.744	-0.056	116	0.217	-0.115
Lactate dehydrogenase (U/L)	154	0.622	-0.040	34	0.985	-0.003	116	0.586	-0.051

Bold font indicates statistically significant *P* values. Abbreviations: NT-pro BNP: N-Terminal pro-B-type natriuretic peptide; eGFR: the estimated glomerular filtration rate; MELD: model for end-stage liver disease.

**Table 4:** Multivariate linear regression analysis of factors associated with the NT-pro BNP level.

Factors	No. Pts	Age-adjusted	
		*B*-coefficient (SE)	*P* value
Age	158	0.302 (0.008)	**<0.001**
Gender	158	0.106 (0.214)	0.137
Acute decompensating events	158	0.432 (0.229)	**<0.001**
Ascites	158	0.175 (0.195)	**0.014**
Acute gastrointestinal hemorrhage	158	0.309 (0.183)	**<0.001**
ACLF	158	0.218 (0.384)	**<0.001**

Bold font indicates statistically significant *P* values. Serum NT-pro BNP concentrations were log_10_-transformed in order to normalize their distribution. Abbreviations: NT-pro BNP: N-Terminal pro-B-type natriuretic peptide; SE: standard error; ACLF: acute-on-chronic liver failure.

## Data Availability

The data used to support the findings of this study are available from the corresponding author upon reasonable request.

## Supplementary Materials

Supplementary Materials Supplementary Table 1: a table showing partial correlation analysis of the hs-cTnT level in cirrhotic patients, cirrhotic patients with and without decompensation using *P* value, correlation coefficient parameter. Supplementary Table 2: a table showing partial correlation analysis of the CK level in cirrhotic patients using *P* value, correlation coefficient parameter. Supplementary Table 3: a table showing partial correlation analysis of the CK-MB level in cirrhotic patients using *P* value, correlation coefficient parameter. Supplementary Table 4: a table showing partial correlation analysis of the LDH level in cirrhotic patients using *P* value, correlation coefficient parameter. Supplementary Table 5: a table showing multivariate linear regression analysis of factors, such as age, gender, acute decompensation, ascites, acute gastrointestinal hemorrhage, and ACLF, associated with the hs-cTnT level in cirrhotic patients using *P* value, *B*-coefficient parameter. Supplementary Table 6: a table showing multivariate linear regression analysis of factors, such as age, gender, acute decompensation, ascites, acute gastrointestinal hemorrhage, and ACLF, associated with CK, CK-MB, and LDH levels in cirrhotic patients using *P* value, *B*-coefficient parameter.
