# Supplementary material for: Correlation of Serum Cardiac Markers with Acute Decompensating Events in Liver Cirrhosis
Source: Gastroenterol Res Pract. 2020 Sep 24;2020:4019289. doi: 10.1155/2020/4019289 (PMC7532360; doi:10.1155/2020/4019289)
Supplement: Supplementary Materials — Supplementary Table 1: a table showing partial correlation analysis of the hs-cTnT level in cirrhotic patients, cirrhotic patients with and without decompensation using P value, correlation coefficient parameter. Supplementary Table 2: a table showing partial correlation analysis of the CK level in cirrhotic patients using P value, correlation coefficient parameter. Supplementary Table 3: a table showing partial correlation analysis of the CK-MB level in cirrhotic patients using P value, correlation coefficient parameter. Supplementary Table 4: a table showing partial correlation analysis of the LDH level in cirrhotic patients using P value, correlation coefficient parameter. Supplementary Table 5: a table showing multivariate linear regression analysis of factors, such as age, gender, acute decompensation, ascites, acute gastrointestinal hemorrhage, and ACLF, associated with the hs-cTnT level in cirrhotic patients using P value, B-coefficient parameter. Supplementary Table 6: a table showing multivariate linear regression analysis of factors, such as age, gender, acute decompensation, ascites, acute gastrointestinal hemorrhage, and ACLF, associated with CK, CK-MB, and LDH levels in cirrhotic patients using P value, B-coefficient parameter. [file 4019289.f1.docx]

| **Supplementary table 1. Partial correlation analysis of hs-cTnT level in cirrhosis** | | | | | | | | | | | |
| --- | --- | --- | --- | --- | --- | --- | --- | --- | --- | --- | --- |
| **Variables** | **All liver cirrhosis** | | |  | **Without decompensation** | | |  | **With decompensation** | | |
|  | **No. Pts** | **P value** | **Correlation coefficient** |  | **No. Pts** | **P value** | **Correlation coefficient** |  | **No. Pts** | **P value** | **Correlation coefficient** |
| Age (years) | / | Controlling | / |  | / | Controlling | / |  | / | Controlling | / |
| Sex (Male/Female), n (%) | / | Controlling | / |  | / | Controlling | / |  | / | Controlling | / |
| Red blood cell (10^12^/L) | 166 | **<0.001** | -0.303 |  | 38 | 0.514 | 0.106 |  | 124 | **<0.001** | -0.311 |
| Hemoglobin (g/L) | 166 | **0.001** | -0.245 |  | 38 | 0.647 | 0.075 |  | 124 | **0.005** | -0.248 |
| Hematocrit (%) | 166 | **<0.001** | -0.269 |  | 38 | 0.646 | 0.075 |  | 124 | **0.002** | -0.273 |
| White blood cell (10^9^/L) | 166 | 0.369 | 0.070 |  | 38 | 0.980 | -0.004 |  | 124 | 0.490 | 0.062 |
| Platelets count (10^9^/L) | 166 | 0.814 | 0.018 |  | 38 | 0.667 | 0.070 |  | 124 | 0.837 | 0.018 |
| Total bilirubin (μmol/L) | 166 | 0.661 | -0.034 |  | 38 | 0.893 | 0.022 |  | 124 | 0.548 | -0.054 |
| Direct bilirubin (μmol/L) | 166 | 0.777 | -0.022 |  | 38 | 0.673 | 0.069 |  | 124 | 0.641 | -0.042 |
| Alanine aminotransferase (U/L) | 166 | 0.705 | -0.029 |  | 38 | 0.829 | 0.035 |  | 124 | 0.791 | -0.024 |
| Aspartate aminotransferase (U/L) | 166 | 0.713 | -0.029 |  | 38 | 0.681 | 0.067 |  | 124 | 0.726 | -0.032 |
| Alkaline phosphatase (U/L) | 166 | 0.380 | -0.068 |  | 38 | 0.236 | 0.192 |  | 124 | 0.342 | -0.085 |
| Gamma-glutamyl transpeptidase (U/L) | 166 | 0.668 | -0.033 |  | 38 | 0.361 | 0.148 |  | 124 | 0.589 | -0.049 |
| Albumin (g/L) | 166 | **0.010** | -0.199 |  | 38 | 0.177 | -0.218 |  | 124 | **0.042** | -0.181 |
| Blood urea nitrogen (mmol/L) | 166 | **0.013** | 0.191 |  | 38 | 0.386 | -0.141 |  | 124 | **0.044** | 0.180 |
| Creatinine (μmol/L) | 166 | 0.153 | 0.111 |  | 38 | 0.876 | -0.026 |  | 124 | 0.257 | 0.102 |
| eGFR (ml/min/1.73m^2^) | 166 | 0.140 | -0.114 |  | 38 | 0.982 | 0.004 |  | 124 | 0.186 | -0.119 |
| Sodium (mmol/L) | 166 | 0.979 | 0.002 |  | 38 | 0.934 | 0.013 |  | 124 | 0.803 | 0.022 |
| Prothrombin time (second) | 166 | 0.762 | 0.024 |  | 38 | 0.324 | -0.160 |  | 124 | 0.961 | 0.004 |

| Activated partial thromboplastin time (second) | 166 | 0.550 | 0.046 |  | 38 | 0.449 | 0.123 |  | 124 | 0.536 | 0.056 |
| --- | --- | --- | --- | --- | --- | --- | --- | --- | --- | --- | --- |
| International normalized ratio | 166 | 0.761 | 0.024 |  | 38 | 0.309 | -0.165 |  | 124 | 0.962 | 0.004 |
| D-dimer (mg/L) | 163 | 0.481 | 0.055 |  | 37 | 0.074 | -0.289 |  | 122 | 0.744 | 0.030 |
| High-sensitivity C-reactive protein (mg/L) | 165 | 0.154 | 0.111 |  | 37 | 0.700 | -0.064 |  | 124 | 0.291 | 0.095 |
| MELD score | 166 | 0.898 | 0.010 |  | 38 | 0.442 | -0.125 |  | 124 | 0.855 | -0.016 |
| Child-Pugh score | 166 | 0.289 | 0.082 |  | 38 | 0.582 | -0.090 |  | 124 | 0.655 | 0.040 |
| NT-pro BNP (pg/ml) | 151 | **0.004** | 0.229 |  | 34 | 0.062 | -0.315 |  | 113 | **0.026** | 0.208 |
| Creatine kinase (U/L) | 166 | 0.908 | -0.009 |  | 38 | 0.759 | 0.050 |  | 124 | 0.810 | -0.022 |
| Creatine kinase MB (U/L) | 166 | 0.431 | -0.061 |  | 38 | 0.654 | -0.073 |  | 124 | 0.458 | -0.067 |
| Lactate dehydrogenase (U/L) | 166 | 0.963 | -0.004 |  | 38 | 0.929 | -0.015 |  | 124 | 0.881 | -0.014 |
| Bold font indicates statistically significant P values. Abbreviations: hs-cTnT, High-sensitivity cardiac troponin T; eGFR, the estimated glomerular filtration rate; MELD, model for end-stage liver disease; NT-pro BNP, N-Terminal pro-B-Type natriuretic peptide. | | | | | | | | | | | |

| **Supplementary table 2. Partial correlation analysis of CK level in cirrhosis** | | | |
| --- | --- | --- | --- |
| **Variables** | **CK** | | |
|  | **No. Pts** | **P value** | **Correlation coefficient** |
| Age (years) | / | Controlling | / |
| Sex (Male/Female), n (%) | / | Controlling | / |
| Red blood cell (10^12^/L) | 170 | 0.571 | 0.044 |
| Hemoglobin (g/L) | 170 | 0.168 | 0.105 |
| Hematocrit (%) | 170 | 0.223 | 0.093 |
| White blood cell (10^9^/L) | 170 | 0.321 | -0.076 |
| Platelets count (10^9^/L) | 170 | 0.101 | -0.125 |
| Total bilirubin (μmol/L) | 170 | 0.578 | -0.043 |
| Direct bilirubin (μmol/L) | 170 | 0.617 | -0.038 |
| Alanine aminotransferase (U/L) | 170 | 0.505 | -0.051 |
| Aspartate aminotransferase (U/L) | 170 | 0.870 | -0.013 |
| Alkaline phosphatase (U/L) | 170 | 0.431 | -0.060 |
| Gamma-glutamyl transpeptidase (U/L) | 170 | 0.166 | 0.106 |
| Albumin (g/L) | 170 | **0.041** | -0.156 |
| Blood urea nitrogen (mmol/L) | 170 | 0.580 | 0.042 |
| Creatinine (μmol/L) | 170 | 0.409 | -0.063 |
| eGFR (ml/min/1.73m^2^) | 170 | 0.643 | 0.036 |
| Sodium (mmol/L) | 170 | **0.046** | 0.153 |
| Prothrombin time (second) | 170 | 0.885 | -0.011 |
| Activated partial thromboplastin time (second) | 170 | 0.246 | -0.089 |
| International normalized ratio | 170 | 0.958 | -0.004 |
| D-dimer (mg/L) | 166 | 0.664 | -0.034 |
| High-sensitivity C-reactive protein (mg/L) | 169 | 0.572 | 0.043 |
| MELD score | 170 | 0.572 | -0.043 |
| Child-Pugh score | 170 | 0.392 | 0.066 |
| NT-pro BNP (pg/ml) | 154 | 0.653 | -0.036 |
| High-sensitivity cardiac troponin T (ng/ml) | 166 | 0.908 | -0.009 |
| Creatine kinase MB (U/L) | 170 | **<0.001** | 0.454 |
| Lactate dehydrogenase (U/L) | 170 | **0.002** | 0.239 |
| Bold font indicates statistically significant P values. Abbreviations: CK, Creatine kinase; eGFR, the estimated glomerular filtration rate; MELD, model for end-stage liver disease; NT-pro BNP, N-Terminal pro-B-Type natriuretic peptide. | | | |

| **Supplementary table 3. Partial correlation analysis of CK-MB level in cirrhosis** | | | |
| --- | --- | --- | --- |
| **Variables** | **CK-MB** | | |
|  | **No. Pts** | **P value** | **Correlation coefficient** |
| Age (years) | / | Controlling | / |
| Sex (Male/Female), n (%) | / | Controlling | / |
| Red blood cell (10^12^/L) | 170 | 0.197 | 0.099 |
| Hemoglobin (g/L) | 170 | 0.146 | 0.111 |
| Hematocrit (%) | 170 | 0.157 | 0.108 |
| White blood cell (10^9^/L) | 170 | **0.001** | -0.242 |
| Platelets count (10^9^/L) | 170 | **0.003** | -0.225 |
| Total bilirubin (μmol/L) | 170 | 0.139 | -0.113 |
| Direct bilirubin (μmol/L) | 170 | 0.106 | -0.124 |
| Alanine aminotransferase (U/L) | 170 | 0.259 | -0.087 |
| Aspartate aminotransferase (U/L) | 170 | 0.181 | -0.103 |
| Alkaline phosphatase (U/L) | 170 | 0.695 | -0.030 |
| Gamma-glutamyl transpeptidase (U/L) | 170 | 0.312 | -0.078 |
| Albumin (g/L) | 170 | 0.890 | -0.011 |
| Blood urea nitrogen (mmol/L) | 170 | 0.350 | 0.072 |
| Creatinine (μmol/L) | 170 | 0.829 | -0.017 |
| eGFR (ml/min/1.73m^2^) | 170 | 0.882 | -0.011 |
| Sodium (mmol/L) | 170 | **0.046** | 0.152 |
| Prothrombin time (second) | 170 | 0.408 | -0.063 |
| Activated partial thromboplastin time (second) | 170 | 0.273 | -0.084 |
| International normalized ratio | 170 | 0.418 | -0.062 |
| D-dimer (mg/L) | 166 | 0.169 | -0.107 |
| High-sensitivity C-reactive protein (mg/L) | 169 | 0.078 | -0.135 |
| MELD score | 170 | 0.078 | -0.135 |
| Child-Pugh score | 170 | 0.392 | -0.066 |
| NT-pro BNP (pg/ml) | 154 | 0.209 | -0.101 |
| High-sensitivity cardiac troponin T (ng/ml) | 166 | 0.431 | -0.061 |
| Creatine kinase (U/L) | 170 | **<0.001** | 0.454 |
| Lactate dehydrogenase (U/L) | 170 | **0.027** | 0.169 |
| Bold font indicates statistically significant P values. Abbreviations: CK-MB, Creatine kinase MB; eGFR, the estimated glomerular filtration rate; MELD, model for end-stage liver disease; NT-pro BNP, N-Terminal pro-B-Type natriuretic peptide. | | | |

| **Supplementary table 4. Partial correlation analysis of LDH level in cirrhosis** | | | |
| --- | --- | --- | --- |
| **Variables** | **LDH** | | |
|  | **No. Pts** | **P value** | **Correlation coefficient** |
| Age (years) | / | Controlling | / |
| Sex (Male/Female), n (%) | / | Controlling | / |
| Red blood cell (10^12^/L) | 170 | 0.625 | 0.038 |
| Hemoglobin (g/L) | 170 | **0.018** | 0.180 |
| Hematocrit (%) | 170 | **0.022** | 0.174 |
| White blood cell (10^9^/L) | 170 | **0.010** | 0.195 |
| Platelets count (10^9^/L) | 170 | 0.059 | 0.144 |
| Total bilirubin (μmol/L) | 170 | **<0.001** | 0.383 |
| Direct bilirubin (μmol/L) | 170 | **<0.001** | 0.380 |
| Alanine aminotransferase (U/L) | 170 | **<0.001** | 0.264 |
| Aspartate aminotransferase (U/L) | 170 | **<0.001** | 0.387 |
| Alkaline phosphatase (U/L) | 170 | 0.089 | 0.130 |
| Gamma-glutamyl transpeptidase (U/L) | 170 | **0.009** | 0.199 |
| Albumin (g/L) | 170 | **0.004** | -0.219 |
| Blood urea nitrogen (mmol/L) | 170 | 0.836 | 0.016 |
| Creatinine (μmol/L) | 170 | 0.906 | 0.009 |
| eGFR (ml/min/1.73m^2^) | 170 | 0.616 | 0.038 |
| Sodium (mmol/L) | 170 | 0.058 | -0.145 |
| Prothrombin time (second) | 170 | **0.004** | 0.220 |
| Activated partial thromboplastin time (second) | 170 | 0.066 | 0.140 |
| International normalized ratio | 170 | **0.004** | 0.221 |
| D-dimer (mg/L) | 166 | **0.001** | 0.255 |
| High-sensitivity C-reactive protein (mg/L) | 169 | 0.081 | 0.134 |
| MELD score | 170 | **<0.001** | 0.336 |
| Child-Pugh score | 170 | **<0.001** | 0.353 |
| NT-pro BNP (pg/ml) | 154 | 0.622 | -0.040 |
| High-sensitivity cardiac troponin T (ng/ml) | 166 | 0.963 | -0.004 |
| Creatine kinase (U/L) | 170 | **0.002** | 0.239 |
| Creatine kinase MB (U/L) | 170 | **0.027** | 0.169 |
| Bold font indicates statistically significant P values. Abbreviations: LDH, Lactate dehydrogenase; eGFR, the estimated glomerular filtration rate; MELD, model for end-stage liver disease; NT-pro BNP, N-Terminal pro-B-Type natriuretic peptide. | | | |

| **Supplementary table 5. Multivariate linear regression analysis of factors associated with hs-cTnT level** | | | |
| --- | --- | --- | --- |
| **Factors** | **No. Pts** | **Age-adjusted** | |
|  |  | **B-coefficient (SE)** | **P-value** |
| Age | 170 | 0.272 (0.006) | **<0.001** |
| Gender | 170 | -0.113 (0.147) | 0.14 |
| Acute decompensation | 170 | 0.187 (0.150) | **0.012** |
| Ascites | 170 | 0.100 (0.134) | 0.187 |
| Acute gastrointestinal hemorrhage | 170 | 0.258 (0.128) | **<0.001** |
| ACLF | 170 | 0.061 (0.289) | 0.408 |
| Bold font indicates statistically significant P values. Serum hs-cTnT concentrations were log_10_-transformed in order normalize their distribution. Abbreviations: hs-cTnT, High-sensitivity cardiac troponin T; ACLF, acute-on-chronic liver failure. | | | |

| **Supplementary table 6. Multivariate linear regression analysis of factors associated with CK, CK-MB and LDH level** | | | | | | | | | | | |
| --- | --- | --- | --- | --- | --- | --- | --- | --- | --- | --- | --- |
| **Decompensated events** | **CK** | | |  | **CK-MB** | | |  | **LDH** | | |
|  | **No. Pts** | **Gender-adjusted** | |  | **No. Pts** | **B-coefficient (SE)** | **P-value** |  | **No. Pts** | **Gender-adjusted** | |
|  |  | **B-coefficient (SE)** | **P-value** |  |  |  |  |  |  | **B-coefficient (SE)** | **P-value** |
| Age | 170 | -0.116 (0.005) | 0.136 |  | 170 | -0.152 (0.004) | 0.056 |  | 170 | 0.077 (0.002) | 0.323 |
| Gender | 170 | -0.190 (0.112) | **0.016** |  | 170 | 0.053 (0.115) | 0.506 |  | 170 | 0.166 (0.050) | **0.034** |
| Acute decompensated events | 170 | -0.004 (0.127) | 0.954 |  | 170 | -0.018 (0.120) | 0.819 |  | 170 | 0.085 (0.052) | 0.268 |
| Ascites | 170 | -0.067 (0.115) | 0.398 |  | 170 | -0.018 (0.108) | 0.827 |  | 170 | 0.164 (0.044) | **0.028** |
| Acute gastrointestinal hemorrhage | 170 | -0.039 (0.110) | 0.613 |  | 170 | -0.054 (0.104) | 0.491 |  | 170 | -0.185 (0.043) | **0.011** |
| ACLF | 170 | -0.004 (0.238) | 0.954 |  | 170 | -0.071 (0.225) | 0.37 |  | 170 | 0.151 (0.094) | **0.042** |
| Bold font indicates statistically significant P values. Serum CK, CK-MB, LDH concentrations were log_10_-transformed in order normalize their distribution. Abbreviations: CK, creatine kinase; CK-MB, creatine kinase MB; LDH, lactate dehydrogenase; ACLF, acute-on-chronic liver failure. | | | | | | | | | | | |
